# The Effect of Anaesthesia Management with Different Fresh Gas Flows on Cognitive Functions of Geriatric Patients: A Randomized Double-blind Study

**DOI:** 10.4274/TJAR.2022.21630

**Published:** 2023-06-16

**Authors:** Bilge Özge Kılıç, Meltem Savran Karadeniz, Emre Şentürk, Meltem Merve Güler, İbrahim Hakan Gürvit, Zerrin Sungur, Ebru Demirel, Kamil Mehmet Tuğrul

**Affiliations:** 1 Department of Anaesthesiology and Reanimation, İstanbul University İstanbul Faculty of Medicine, İstanbul, Turkey; 2 Department of Neurology, İstanbul University, İstanbul Faculty of Medicine, İstanbul, Turkey

**Keywords:** Agitation, cognitive dysfunction, emergence, geriatric anaesthesia, low flow

## Abstract

**Objective:**

The present study aimed to compare the effects of two different fresh gas flows (FGFs) (0.5 L min^-1^ and 2 L min^-1^) applied during maintenance of anaesthesia on recovery from anaesthesia and early cognitive functions in geriatric patients.

**Methods:**

In this prospective, randomised, double-blind study, sixty patients were divided into two groups according to the amount of FGF. Minimal-flow anaesthesia (0.5 L min^-1^ FGF) was applied to group I and medium-flow anaesthesia (2 L min^-1^ FGF) was applied to group II during maintenance of anaesthesia. Following the termination of inhalation anaesthesia, recovery times were recorded. The evaluation of cognitive functions was performed using the Addenbrooke’s Cognitive Examination (ACE-R).

**Results:**

There was no significant difference between the two groups in terms of demographic characteristics and recovery (*P* > 0.05). There was no significant difference between the two groups in terms of the preoperative day, the first postoperative day, and the third postoperative day; ACE-R scores (*P* > 0.05). In group II, on the third postoperative day ACE-R scores were found to be significantly lower than the preoperative ACE-R scores (*P*=0.04). In group II, third postoperative day ACE-R memory sub-scores (14.53 ± 3.34) were found to be significantly lower than preoperative ACE-R memory sub-scores (15.03 ± 3.57) (*P*=0.04).

**Conclusion:**

In geriatric patients, minimal-flow anaesthesia was not superior to medium-flow anaesthesia in terms of recovery properties and cognitive functions. Keeping in mind that hypoxaemia and changes in anaesthesia levels may occur with the reduction of FGF, both minimal- and medium-flow anaesthesia can be applied with appropriate monitoring without adverse effects on recovery and cognitive functions.

**Main Points**

• With low-flow anaesthesia (LFA), personnel exposure, cost, and greenhouse gas effects are reduced; also, LFA contributes to respiratory physiology. Furthermore, LFA increases the quality of recovery, but if fresh gas flow (FGF) is not increased in the recovery phase, it may cause prolongation of such a phase.

• To avoid prolonged recovery times, closing the vaporiser by increasing the FGF to 6 L min-1 at the end of the operation can provide faster recovery without affecting the recovery quality and without facing the risk of awareness.

• There are not enough studies investigating the effect of different FGFs used in anaesthesia maintenance on early postoperative cognitive dysfunction. Neither minimal-flow anaesthesia nor medium-flow anaesthesia is superior to each other in terms of postoperative cognitive functions in geriatric patients. Both FGFs can be used in the maintenance of anaesthesia in geriatric patients without additional concern for cognitive dysfunction.

## Introduction

Environmental and economic advantages, as well as physiological ones (e.g., increasing the recovery quality, preserving the temperature and humidity of the inhaled gas mixture), have increased the popularity of low-flow anaesthesia (LFA) in recent years.^1^ Modern anaesthesia machines, inspiratory air monitoring, and third-generation inhalation agents have made LFA possible with a fresh gas flow (FGF) of up to 250 mL min^-1^.^2^ Despite this trend, there are few studies examining the relationship between different FGFs and early postoperative cognitive dysfunction (POCD).^3^

Neurocognitive functions include components such as memory, attention and language, which are controlled by certain pathways and centres in the brain.^4^ Since Bedford’s 1955 article, it is well known that the perioperative process causes varying degrees of cognitive dysfunction in elderly patients.^4^ Despite advances in perioperative medical knowledge and techniques, POCD is still associated with increased postoperative complications, prolonged hospitalisation, early retirement, increased cost, and mortality.^5^

Early POCD is seen in 26% of elderly patients in non-cardiac surgeries.^6^ Neuroinflammation and inactivation of the cholinergic system play a key role in the pathophysiology of POCD.^7^ Due to increased peripheral inflammatory responses and an impaired blood-brain barrier, major surgeries and advancing age are the most important risk factors for POCD. However, it has not been shown that any anaesthetic technique currently used is superior to the others.^4^

This study examined whether different FGFs (minimal or medium flows) influence recovery from anaesthesia and early postoperative cognitive functions in geriatric patients. Our primary hypothesis was that early postoperative cognitive function scores would be higher in minimal-flow anaesthesia than in traditional medium-flow anaesthesia. Therefore, our primary outcome was the change in postoperative cognition tests. Our secondary outcomes were eye opening, obeying verbal orders, first movement, extubation times, time to reach the Steward score, recovery agitation, awareness development, and hospital stay.

## Methods

### Study Group and Randomisation

This prospective randomised double-blind study was conducted between October 2017 and February 2018, following İstanbul University, İstanbul Faculty of Medicine Clinical Research Ethics Committee approval (27.10.2017/17) and patient consent. Patients aged ≥60 years scheduled to be operated on under general anaesthesia at the İstanbul University Urology Clinic, with American Society of Anesthesiologists classification I-II-III and an estimated operation time over 2 hours were included. Patients who refused to participate in the study, had neurological or psychiatric diagnoses, had hearing or vision problems, had language barriers, were followed up in the intensive care unit in the early postoperative period, could not perform at least one of the planned neuropsychological tests, or had a mini-mental test score of <26 were excluded from the study.

The participants were randomised and divided into two groups; 0.5 L min^-1^ FGF was applied to group I and 2 L min^-1^ FGF was applied to group II. The randomisation sequence of the study was obtained using a computer programme by a researcher blinded to the FGF level and the neuropsychological tests to be used on patients (https://www.graphpad.com/quickcalcs/randomise1/). The information concerning the group that the patients would be included in was placed in sealed, opaque envelopes. Each sealed envelope was opened just before the induction of anaesthesia. The patients and the clinician who performed the neuropsychological tests were blinded to the anaesthesia method.

### Anaesthesia Management

Electrocardiography, pulse oximetry (SpO_2_), arterial blood pressure oscillometric measurement, bispectral index (BIS), and end-tidal carbon dioxide (EtCO_2_) monitoring were performed on all patients who were admitted to the operating room. The patients were warmed actively during the operation using a heating bed. Induction of anaesthesia was performed with 1 µg kg^-1^ fentanyl, 2 mg kg^-1^ propofol, and 0.6 mg kg^-1^ rocuronium. The age-related desflurane nomogram was used to determine the end-tidal desflurane concentration, which corresponds to the target minimum alveolar concentration (MAC) value of 1.2 after intubation.^8^ The initial setting of the desflurane vaporiser was determined by adding 1% to the value measured on the nomogram. Ventilation parameters were adjusted so that FGF was 4 L min^-1^ with 60% O_2_ and air mixtures. The tidal volume was 6-8 mL kg^-1^. Respiratory frequency was 12-14, and positive end-expiratory pressure was 4-6 cm H_2_O (Dräger Primus^®^). When the MAC reached 1.2, FGF was reduced to 0.5 L min^-1^ in group I and 2 L min^-1^ in group II. Intravenous remifentanil infusion (0.05-0.2 µg kg^-1^ min^-1^) was administered to all patients throughout the operation to provide intraoperative analgesia. Remifentanil infusion was titrated with dose changes of 0.01-0.02 µg kg^-1^ min^-1^ so that BIS values ​​were between 40 and 60. The inhalation of the hypoxic gas mixture and changes in anaesthesia levels were prevented by monitoring the inhaled O_2_ concentrations (FiO_2_) and MAC values ​​throughout the operation. When FiO_2_ dropped below 35%, O_2_ flow increased by 10% of the total gas flow. When FGF was reduced to 0.5 L min^-1^ in group I, the desflurane vaporiser setting was increased by 1% of the FGF volume to prevent superficial anaesthesia levels. When FGF was reduced to 2 L min^-1^ in Group II, no change was made to the desflurane vaporiser setting. Up to 1% desflurane vaporiser setting change was allowed, keeping the MAC values ​​at 1.2 in both groups.

At the end of the operation, FGF was adjusted to 6 L min^-1^ 100% O_2_. 10 min after the end of the operation, 0.5 mg atropine and 1.5 mg neostigmine were administered to each patient. After the end-tidal desflurane concentration was 0% and the BIS value was >80, the patients who met the extubation criteria were extubated. The extubation criteria were as follows: the patient was cooperative, the tidal volume was >6 mL kg^-1^, and the patient was able to raise his head for >5 s. Wake-up time was evaluated with the Steward recovery score (SRS).^9^ Patients with SRS ≥4 were transported to the recovery room. The time it took patients to open their eyes, respond to verbal commands, be extubated, and be transported to the recovery room after the desflurane vaporiser shutdown was recorded.

Hypotension was defined as mean arterial pressure <65 mmHg and hypoxaemia as SpO_2_ <90%. In the case of hypotension, 5 mg ephedrine IV was administered. Arterial blood samples were taken and analysed from all patients at 90-minute intervals, the first one being at the beginning of the operation. Erythrocyte suspension was administered to patients with haemoglobin <8 g dL^-1^. The pre-operative and postoperative blood glucose and sodium values ​​of all patients were recorded.

At the end of the operation, 1 g paracetamol IV and morphine 0.05 mg kg^-1^ IV were administered at 6-h intervals to each patient. 0.03 mg kg^-1^ morphine was administered as an additional analgesic to patients with visual analogue scale (VAS) ≥4.

The Richmond Agitation-Sedation Scale (RASS) was used to evaluate recovery agitation.^10^ Recovery agitation was diagnosed in patients with RASS ≥2 in the recovery room follow-up. The cognitive functions of the patients were evaluated one day before the operation, on the first postoperative day, and on the third postoperative day using the Addenbrooke’s Cognitive Examination (ACE-R). To prevent environmental conditions from affecting the neuropsychological test results, all tests were performed in a quiet room. To prevent the learning effect, three different forms of ACE-R adapted to Turkish society were applied.^11^ Postoperative neuropsychological evaluations of all patients were performed when VAS <4. Awareness during general anaesthesia was questioned with the modified Brice scale on the days ACE-R was administered (Table 1).^12^ According to this scale, patients who stated that they experienced awareness in questions 4 and 5 and those who answered “yes” to question 3 were diagnosed with intraoperative awareness.

### Sample Size Calculation

The G-Power program (version 3.1.9.2, Kiel, Germany) was used to determine the sample size before the study. In the preliminary study, the effect size was determined as 0.7. In this study, which creates a sample at a ratio of 1:1 for both groups, α: 0.05. When 1-β: 0.80 and considering 25% data loss, the aim was to include forty patients for each group, or eighty patients.

### Statistical Analysis

The SPSS 20.0 program (IBM, United States, 1963) was used for statistical analysis. The conformity of the data to the normal distribution was examined with the Kolmogorov-Smirnov test. Normally distributed data were expressed as the mean and standard deviation and compared with independent sample *t*-test. Nominal data were expressed as the number and percentage of cases and compared with the chi-square test (Pearson’s chi-square test and Fisher’s exact test). In-group changes were investigated in repeated measurements, and the single-factor ANOVA test was used. Statistical significance was accepted as *P* < 0.05.

## Results

**Figure 1:**
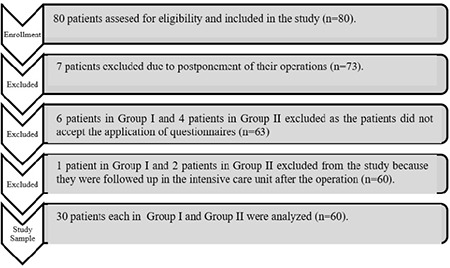
Flowchart of the study.

A total of eighty patients were included in the study. Seven patients who underwent preoperative ACE-R were excluded from the study because of the postponement of their operations. Six patients from group I and four patients from group II were excluded from the study because they did not accept the application of ACE-R on the first or third postoperative day. One patient from group I and two patients from group II was excluded from the study because they were followed up in the intensive care unit after the operation. The study was completed with thirty patients in both groups (Figure 1).

No significant difference was observed between the two groups in terms of gender, age, comorbid systemic diseases, education, occupation, smoking, multiple drug use, type of anaesthesia applied in previous surgeries, duration of operation, duration of anaesthesia and hospital stay (*P* > 0.05) (Table 2). There was no statistically significant difference between the two groups in terms of pre-operative and postoperative serum glucose and sodium values (*P* > 0.05) (Table 3).

There was no statistically significant difference between the two groups in terms of intraoperative ephedrine requirement (33.3%; 26.6%) and intraoperative transfusion requirement (0%; 3.3%) (*P* > 0.05). The proportion of patients with FiO_2_ <35% was found to be statistically significantly higher in group I (53.3%) than in group II (26.6%) (*P*=0.04) (Table 3). Hypoxaemia did not develop in any patients in the two groups.

There was no statistically significant difference between the two groups in terms of eye opening, obeying verbal orders, first movement, extubation, and Steward score ≥4 (*P* > 0.05) (Table 4). According to RASS, recovery agitation did not develop in any patients in the two groups. According to the modified Brice scale, awareness did not develop in any patients in the two groups. There was no significant difference between the two groups in terms of baseline, intraoperative 30^th^, 60^th^, 90^th^, and 120^th^ min BIS values (*P* > 0.05) (Table 5).

The ACE-R scores and ACE-R subparameter scores of the patients in groups I and II are shown in Table 6. There was no statistical difference between the two groups in terms of ACE-R scores applied on the pre-operative day, the first postoperative day, and the third postoperative day (*P* > 0.05). The ACE-R total scores of the patients in group I were found to be statistically similar on the pre-operative day, the first postoperative day, and the third postoperative day (*P* > 0.05). In group II, the third postoperative day total ACE-R scores (75.6 ± 7.3) were found to be statistically significantly lower than the pre-operative ACE-R scores (76.1 ± 10.04) (*P*=0.04). In group II, there was no statistically significant difference between the ACE-R scores obtained on the first postoperative day and the ACE-R scores obtained on the pre-operative day and the third postoperative day (*P* > 0.05). When the subsections of ACE-R were evaluated, there was no statistically significant difference between the two groups (*P* > 0.05). In group II, the third postoperative day memory scores (14.53 ± 3.3) were found to be statistically significantly lower than the pre-operative memory scores (15.03 ± 3.5) (*P*=0.04). No statistically significant difference was observed among other subsections of ACE-R administered at different times in group I (*P* > 0.05).

## Discussion

Our study showed that medium- and minimal-flow anaesthesia were not superior to each other in terms of recovery criteria and postoperative early cognitive functions among geriatric patients undergoing elective urological surgery. Furthermore, although it was not clinically significant in patients who underwent medium-flow anaesthesia, the general cognitive and memory scores on the third postoperative day decreased by about half a point compared to the pre-operative values.

Technological and pharmacological developments have made it possible to reduce the O_2_ flow during the maintenance of anaesthesia to the basal metabolic needs of patients. The analysis of respiratory gases and the temporary increase of FGF has largely prevented the problems that may be encountered during LFA.^1^ LFA reduces the consumption of inhalation anaesthetics, thus reducing cost, personnel exposure, and greenhouse effects. It also contributes to respiratory functions by preventing heat and moisture loss through respiratory gases during general anaesthesia.

A low FGF prevents rapid changes in brain and alveolar agent concentrations during the termination of anaesthesia, improving recovery quality but prolonging recovery time.^1,13^ In the study of Jeong et al.^14^, after desflurane anaesthesia was applied with different FG, recovery times were found to be longer in patients who were administered 2 L min^-1^ FGF (17.6 min) compared to 4-6 L min^-1^ FGF (9.9 min, 9.1 min, respectively). Recovery agitation or awareness did not develop in any of the patients in this study. It was concluded that if the desflurane vaporiser is turned off at the prescribed time according to the FGF applied before the end of the operation, it is possible to use LFA in the recovery phase without loss of time. However, 20% of awareness cases in anaesthesia occur during the recovery period.^15^ In our study, the desflurane vaporiser was turned off after the operation was completed to avoid the risk of awareness in the last phase of the operation. The recovery times of our patients, whose FGF was increased to 6 L min^-1^ after the operation and whose time constant was shortened, were consistent with those of Jeong et al.^14^ 4-6 L min^-1^ FGF-applied patients, but they were faster than those in whom low flow was applied. Furthermore, none of our patients experienced awareness or recovery agitation. These data show that a quality recovery can be achieved without complications (e.g., awareness and agitation) with intraoperative depth of anaesthesia monitoring and closing of the vaporiser by increasing FGF up to 6 L min^-1^ after the operation is completed. Especially when monitoring methods such as BIS are not used, the vaporiser should not be turned off before the operation is completed.

In a randomised study, FGF was increased to 6 L min^-1^ during the recovery period of patients who underwent minimal-flow (0.5 L min^-1^), low-flow (1 L min^-1^) and medium-flow (2 L min^-1^) anaesthesia, and no significant difference was found in terms of recovery duration among the groups.^16^ These results show that recovery times are related to the adjusted FGF during the recovery period rather than the adjusted FGF during anaesthesia maintenance.

Early POCD is a serious complication associated with significant morbidity and mortality and it is seen in more than a quarter of post-operative geriatric patients. Geriatric surgery candidates constitute the riskiest patients in terms of postoperative cognitive dysfunction. Neuropsychological tests are essential to detect perioperative cognitive performance changes.^4^ ACE-R, which was validated in our study, can be used to measure general cognitive performance and has different forms to prevent the learning effect.^11^

Chan et al.^17^ showed that recovery from anaesthesia is faster and the incidence of early and late postoperative cognitive dysfunctions is reduced in patients with intraoperative BIS monitoring. However, a direct relationship between rapid and smooth recovery and POCD has not been demonstrated. In our study, we examined the relationship between minimal- and medium-flow anaesthesia methods and recovery and postoperative cognitive functions.

According to the results of our study, there was no difference in the ACE-R scores of patients who were administered medium- and minimal-flow anaesthesia at all times. Among the in-group ACE-R score changes according to time, we found that the general ACE-R and memory scores were lower on the third postoperative day compared with the pre-operative scores in only the medium-flow anaesthesia group. However, as in the criticism of Chandrasekhar et al.^18^, we think that the 0.5-point difference, which was found to be statistically significant, is not clinically significant.^19^ Our data show that the two different FGFs are not superior to each other in preventing POCD. As far as we know, there is only one study examining the relationship between different FGF currents and POCD. Muslu et al.^3^ found no significant difference in POCD between the LFA method (1 L min^-1^ FGF) and the medium-flow anaesthesia method (4 L min^-1^ FGF) in laparoscopic cholecystectomy cases, where sevoflurane was used for anaesthesia maintenance. Since the neuropsychological tests were performed only on the first postoperative day and four times in total, a significant learning effect was experienced, similar to the patients in our study who underwent minimal-flow anaesthesia. Evaluation of tests at close intervals, patients’ familiarity with the modified test format, and learning the answers to some questions may result in higher results in repeated tests in the postoperative period.

There is no significant difference between the two groups of our study in terms of demographic characteristics, medical history, surgical history, perioperative results and laboratory parameters, which have been shown in different studies to have an effect on advanced age and postoperative cognitive performance.^20^

During LFA, it is essential to monitor the concentrations of gases in the exhaled air to prevent the patient from inhaling a hypoxic gas mixture and to maintain adequate depth of anaesthesia. Hypoxaemia, insufficient anaesthesia, and deep anaesthesia levels are among the risk factors that have been shown to be associated with POCD.^17,21^ In our study, standard anaesthesia management and monitoring were applied to prevent these factors from affecting the perioperative cognitive function scores. When the breathing air O_2_ concentration in LFA falls below 30%, the FGF O_2_ concentration should be increased by 10%.^22^ Since hypoxaemia may develop, albeit rarely, when the intraoperative FiO_2_ is ≤30, for ethical reasons, the intervention was performed on elderly patients who were more sensitive to the negative effects of anaesthesia and surgery when the FiO_2_ was ≤35%.^23^ According to the results of our study, the need to increase the O_2_ concentration of FGF in the minimal-flow anaesthesia group was significantly higher than in the medium-flow anaesthesia group, but hypoxaemia did not develop in either group. As a result, with close follow-up and appropriate monitoring, LFA can be applied in elderly patients without adversely affecting oxygenation. Park et al.^24^ showed that FiO_2_ was lower in patients who underwent 0.5 L min^-1^ FGF in laparoscopic urological surgeries that were expected to last longer than 6 h, which confirms our results compared to patients who underwent 4 L min^-1^ FGF at every stage of the operation.

Despite a lack of evidence, it is thought that the risk of awareness is higher in LFA. Since MAC has an effect on the spinal cord rather than the brain and is completely independent from the effects of other intravenous agents, it is insufficient in patients with awareness risk.^25^ In our study, certain inhalation anaesthesia protocols were applied to both groups, and the average BIS values ​​were kept at around 50 in all patients. Awareness did not develop in any patient. In addition to the depth of intraoperative anaesthesia, inadequate postoperative pain control is associated with postoperative cognitive dysfunction.^26^ As carried out in our study, the application of standard analgesia protocols and the predetermination of the treatment to be applied when pain scores are high may be beneficial in preventing postoperative cognitive dysfunction.^6,27^ In our study, all the neuropsychological tests were applied when the patient VAS was <4. However, a recent study found the threshold VAS value for POCD to be 2.6 with high specificity and sensitivity.^26^

Our study has several limitations. First, only the effects of LFA on early postoperative cognitive function were shown in our study, and long-term neuropsychological tests were not applied. Second, targeting lower VAS values ​​could improve the standardisation of the two groups, since VAS =4, which we accepted as the threshold value at the time of our study, has been shown to be associated with early POCD recent studies. Third, although we have followed up on VAS in our patients, their VAS values ​​and analgesic needs have not been recorded. Therefore, the sedation levels due to the use of additional morphine may have affected our results. Fourth, in the presence of a larger sample size, a significant difference could be revealed in terms of recovery times and perioperative cognitive dysfunctions, which were quite close to each other in our study. Fifth, the fact that neuromuscular monitoring and monitoring of inhalational anaesthetic agent consumption, which are additional monitoring methods, were not used constitutes another limitation of our study.

## Conclusion

This study has shown that minimal- and medium-flow anaesthesia are not superior to each other in terms of recovery times and perioperative cognitive dysfunction in geriatric patients. Bearing in mind the complications (e.g., hypoxaemia and awareness) that may develop during the maintenance of anaesthesia in geriatric patients, both minimal- and medium-flow anaesthesia can be safely applied without adverse effects on recovery and perioperative cognitive functions, accompanied by monitoring of breathing air and depth of anaesthesia.

## Figures and Tables

**Table 1:**
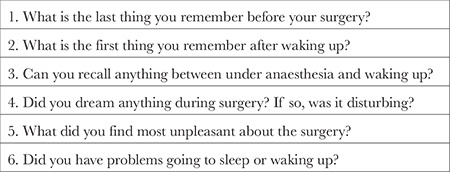
Modified Brice Questionnaire

**Table 2:**
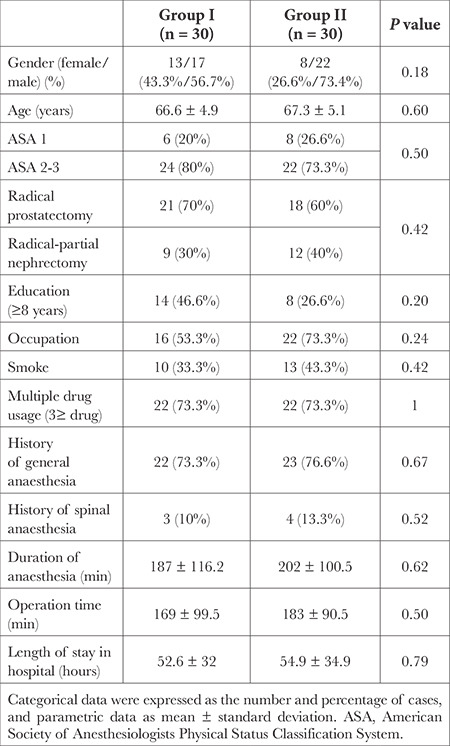
Demographic and Operational Data of the Patients

**Table 3:**
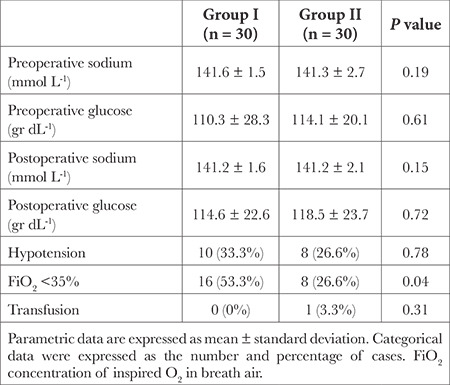
Perioperative Monitoring and Laboratory Data of the Patients

**Table 4:**
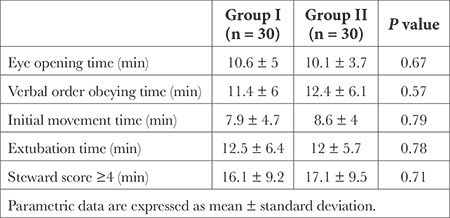
Comparison of Recovery Times of Patients

**Table 5:**
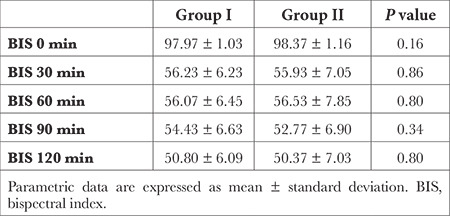
Intraoperative BIS Values

**Table 6:**
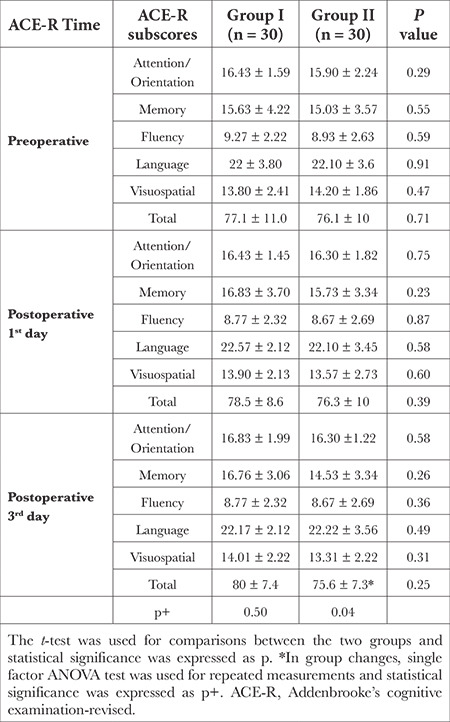
Comparison of Preoperative, Postoperative 1^st^ and 3^rd^ Day ACE-R Total and Sub-Scores Between the Two Groups and Comparison of the Variation of ACE-R Total Scores Over Time within the Group
